# Prospective observational study to evaluate the persistence of treatment with denosumab in patients with bone metastases from solid tumors in routine clinical practice: final analysis

**DOI:** 10.1007/s00520-019-04988-7

**Published:** 2019-07-26

**Authors:** Ferdinand Haslbauer, Andreas Petzer, Martin Safanda, Antoaneta Tomova, Miriam Porubska, Zoltán Bajory, Daniela Niepel, Christine Jaeger, Katja Bjorklof, Dmitry Kalinin, Richard Greil

**Affiliations:** 1 Abteilung für Innere Medizin, Salzkammergut Klinikum Vöcklabruck, Dr.-Wilhelm-Bock-Straße 1, A-4840 Vöcklabruck, Austria; 2 Ordensklinikum Linz Barmherzige Schwestern/Elisabethinen, Linz, Austria; 3 Nemocnice Na Homolce, Prague, Czech Republic; 4 Complex Oncology Center Plovdiv EOOD, Plovdiv, Bulgaria; 5 Onkologicky ustav Sv. Alzbety, Bratislava, Slovak Republic; 6 University of Szeged, Szeged, Hungary; 7 Global Medical Affairs, Amgen GmbH, Europe HQ, Rotkreuz, Switzerland; 8 Medical Affairs, Amgen GmbH, Vienna, Austria; 9 Medical Affairs, Amgen (Europe North East), Rotkreuz, Switzerland; 10 Quartesian, Kharkov, Ukraine; 11 Paracelsus Medizinische Privatuniversität, Salzburger Landeskliniken - Universitätsklinikum Salzburg, Salzburg Cancer Research Institute, Salzburg, Austria

**Keywords:** Denosumab, Persistence, Observational study, Bone metastases, Solid tumors

## Abstract

**Purpose:**

In the integrated analysis of phase III head-to-head trials in patients with advanced solid tumors, denosumab demonstrated superiority over zoledronic acid in preventing skeletal-related events (SREs). Regular and continued drug use (persistence) is a precondition of clinical efficacy; persistence in real-life is yet undetermined for denosumab.

**Methods:**

This was a single-arm, prospective, observational, non-interventional study in 598 patients with bone metastases from breast, prostate, lung, or other solid tumors treated with denosumab every four weeks in real-world clinical practice in Austria, Czech Republic, Hungary, Slovakia, and Bulgaria. Persistence was defined as denosumab administration at ≤ 35-day intervals over 24 or 48 weeks, respectively.

**Results:**

Previous SREs were found in 10.9% of patients. 62.6% were persistent over 24 weeks and 40.1% over 48 weeks. The Kaplan-Meier median (95% CI) time to non-persistence was 274.0 days (232.0, 316.0). The most frequent reason for non-persistence was delayed administration. There was a trend towards weaker analgesics over time, with approximately 60% of patients not requiring any analgesics. Serum calcium remained within the normal range throughout the study. Adjudicated osteonecrosis of the jaw was documented in three patients with an incidence per patient-year (95% CI) of 0.012 (0.004, 0.029).

**Conclusions:**

Most patients received denosumab regularly once every four weeks over 24 weeks of treatment. Non-persistence was mainly due to delayed administration. The incidence of adverse drug reactions, especially of osteonecrosis of the jaw, was in line with expectations from previous studies.

**Electronic supplementary material:**

The online version of this article (10.1007/s00520-019-04988-7) contains supplementary material, which is available to authorized users.

## Introduction

Bone metastases represent a frequent complication of cancer, with more than 1.5 million affected patients worldwide [1]. Clinically important skeletal complications are the result of osteoclast-mediated bone destruction [2, 3] often leading to severe pain, decreased quality of life, instability, and neurologic compromise [4].

Denosumab, a fully human monoclonal antibody of the IgG2 subtype, inhibits the receptor activator of nuclear factor κB ligand (RANKL) on bone cells. In its oncological formulation, denosumab is indicated in Europe for the prevention of skeletal-related events (SREs; pathological fracture, radiation to bone, spinal cord compression, or surgery to bone) in adults with advanced malignancies involving the bone and for the treatment of adults and skeletally mature adolescents with giant cell tumor of bone that is unresectable or where surgical resection is likely to result in severe morbidity [5].

In the integrated analysis of three pivotal, phase III head-to-head trials, denosumab was superior in preventing SREs compared with zoledronic acid [6]. Yet, in real-world clinical routine, irregular administration or unplanned interruption or discontinuation of therapy may impact the therapeutic potential of denosumab in comparison with the efficacy demonstrated in controlled clinical trials. As per International Society for Pharmacoeconomics and Outcomes Research (ISPOR) definition, medication compliance refers to the act of conforming to the recommendations made by the provider with respect to timing, dosage, and frequency of medication taking (= percentage of doses taken as prescribed). Medication persistence refers to the act of conforming to a recommendation of continuing treatment for the prescribed length of time (= days medication was taken without exceeding permissible intervals) [7].

The extent to which poor compliance and persistence affect clinical efficacy is a complex issue. From a payer’s perspective, low compliance and/or persistence often works in two directions: they reduce medication costs but subsequently increase health care resource utilization. Although this cannot necessarily be assumed in all settings, a relationship between bone metastasis–related SREs and additional inpatient stays and an increased use of surgical or other procedures has been demonstrated [8]. To date, the availability of real-life data assessing persistence with denosumab in health care settings in the countries of interest is limited. The convenience of a subcutaneous route of administration and the positive risk/benefit profile of denosumab may result in a high persistence not only in controlled clinical trials but also in real-life clinical practice. The objectives of the present study were to obtain relevant information on real-world practice conditions of denosumab use and on persistence with the drug.

## Methods

### Study design

This was a single-arm, prospective, observational, non-interventional, multi-center cohort study in patients with solid tumors and bone metastases in Austria and selected Central and Eastern European (CEE) countries, namely the Czech Republic, Hungary, Slovakia, and Bulgaria. As this was a non-interventional study, no laboratory, diagnostic, or therapeutic procedures other than those performed as part of the patient’s routine care were required. Patients were observed from enrollment (having received the first dose of denosumab as per standard of care within 28 days prior to enrollment) until the last denosumab dose administered up to a maximum of 48 weeks after the first administration plus 30 days of safety follow-up.

### Eligibility criteria

Patients treated with the denosumab formulation XGEVA® (Amgen Europe B.V., Breda, The Netherlands) at a dose of 120 mg subcutaneously once every four weeks, in accordance with the—at time of enrollment—most current version of the European Medicines Agency’s summary of medicinal product characteristics (SmPC) were eligible to participate in this study. In addition, they had to meet the following criteria: adult age (≥ 18 years) at enrollment; a diagnosis of breast, prostate, lung, or other solid tumor with confirmed bone metastasis; an Eastern Cooperative Oncology Group (ECOG) performance status of 0 to 2; administration of the first denosumab dose ever within 28 days prior to enrollment. Patients were excluded: if they had a diagnosis of multiple myeloma (not an approved indication at the time of study conduct), were previously treated for SRE prevention with bisphosphonates or other antiresorptive agents for more than 6 months, were previously treated with radionuclides (e.g., strontium-98, samarium-153, radium-223), were enrolled in an investigational drug trial for the treatment and/or prevention of bone metastases and SREs, or had contraindications to denosumab. Patients in a trial related to the treatment of their underlying cancer or in long-term follow-up studies were eligible.

### Study objectives

The primary objective was to estimate the persistence with denosumab treatment as per routine clinical practice at 24 weeks. Secondary objectives were to estimate the persistence with denosumab at 48 weeks and the time to and reasons of non-persistence, to describe patient demographics, disease characteristics, concomitant anticancer therapy and medical history, and calcium and vitamin D supplementation patterns. Exploratory objectives included the description of pain medication patterns and patient-reported outcomes according to the EQ-5D questionnaire in countries where this was accepted by local authorities and reasons for the choice of denosumab over other options.

### Reporting of adverse drug reactions

Safety data related to denosumab were collected for up to 30 days after the last denosumab dose. Osteonecrosis of the jaw (ONJ) was regarded as an event of special interest. All suspected events of ONJ were documented and reported as serious adverse drug reactions (ADRs), regardless of whether serious criteria could be assigned and whether causal relationship with denosumab established. Any suspected case of ONJ was reviewed by an independent adjudication panel to confirm or reject the ONJ categorization.

### Ethics

This study complied with all relevant national requirements on a country-by-country basis. Written informed consent was obtained from the patient or legally acceptable representative. National ethics committee approval of the protocol and informed consent form was obtained before recruitment of patients or any data collection.

### Statistical analysis

No formal hypothesis was tested. For continuous variables, descriptive statistics including the mean, standard deviation (SD), median, first (Q1) and third (Q3) quartiles, and minimum and maximum values (range) were presented along with 95% two-sided confidence intervals (CIs), where appropriate. Missing values of continuous variables were counted as “Missing”. For categorical variables, the number and percentage of patients in each category were reported. For binary variables, the number and percentage of patients were reported, along with exact two-sided CIs, where appropriate. Missing results were excluded from the calculation of CIs; however, the number and percentage of patients with missing results were given for categorical data. The statistical analyses were based on the full analysis set (FAS), which consisted of the enrolled patients who met eligibility criteria and received at least one dose of denosumab. The Kaplan-Meier method and Cox proportional hazards model were used to analyze the data for time to non-persistence. For statistical analysis, the SAS System 9.4 was used.

“Persistence” measured the regularity and duration of continuous use of denosumab and was defined as continuous use from the first administration without exceeding a maximum permissible 35-day gap (4 weeks plus 7 days) until the last date of administration (discontinuation date), disenrollment (due to death or loss to follow-up), or end of the study period. Time to non-persistence was calculated as the time between the first injection and the last injection received during the period where the patient was still classified as persistent plus 28 days. When the predefined time window between injections of a maximum of 35 days was exceeded, a drop-down window of reasons for dose interruption opened in the electronic case report form, which were documented as reasons for non-persistence. Additionally, the reason “violation of permissible time window” was documented automatically when the predefined time window of a maximum of 35 days was exceeded based on the documented dates of administration.

## Results

### Patient disposition

**Fig. 1:**
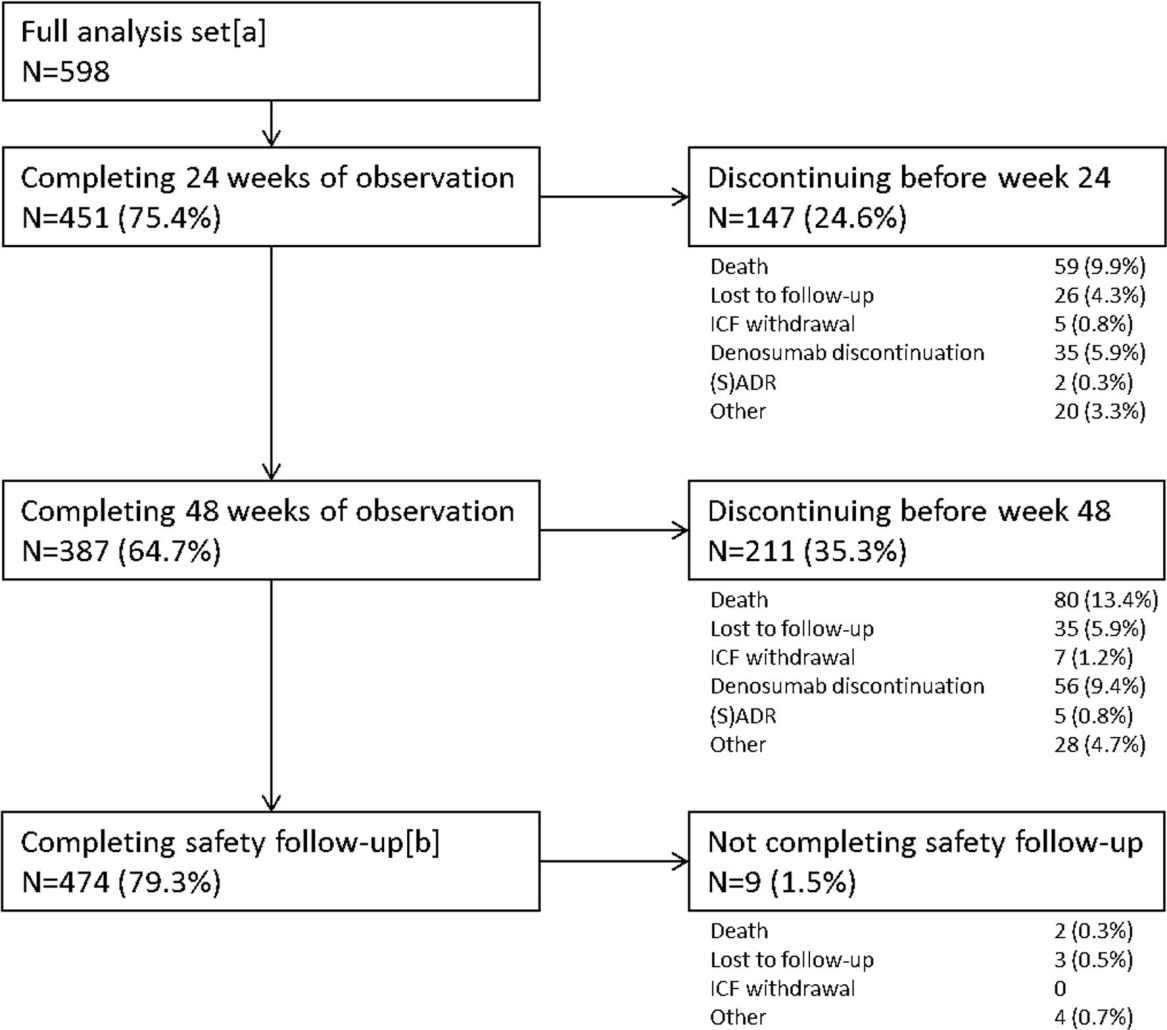
Patient disposition. [a] 634 patients were enrolled; 319 from Austria, 130 from Bulgaria, 109 from the Czech Republic, 58 from Slovakia, and 18 from Hungary. Thirty-six patients were excluded from the analysis. The reasons for exclusion from analysis were violation of inclusion or exclusion criteria, erroneous double entry in the database, or entry by mistake, e.g., erroneously entering a training data set in the real database instead of the training database. [b] Only patients who did not die and were not lost to follow-up are included in this section

A total of 598 patients (FAS) were analyzed (Fig. 1; Table S1, online supplemental material). Of these, 294 patients were from Austria, 130 from Bulgaria, 103 from the Czech Republic, 54 from Slovakia, and 17 from Hungary.

Of 598 patients initiated with denosumab, 451 (75.4%) completed 24 weeks of observation and 147 (24.6%) discontinued prematurely. The main reasons for premature study discontinuation were death (9.9%, *n* = 59), denosumab discontinuation (5.9%, *n* = 35), and loss to follow-up (4.3%, *n* = 26). At week 48, 387 (64.7%) were still under observation and 211 (35.3%) had discontinued the study prematurely. The median (Q1, Q3) duration of study-related observation was 48 weeks (27.3, 49.9). After the end of the study-related observation period, 379 patients (63.4%) continued denosumab treatment. Overall, 91 patients (15.4%) discontinued denosumab, 56 patients during observation and 35 after the end of observation. The documented reasons for discontinuation of denosumab were physician’s decision (*n* = 29, 4.8%), patient’s decision (*n* = 28, 4.7% of FAS), (S)ADRs (*n* = 8, 1.3%: peripheral edema, hypocalcemia, hypophosphatemia, osteonecrosis, or cellulitis), switch to other antiresorptive drugs (*n* = 5, 0.8%), or other reasons (*n* = 21, 3.5%). The overall number of deaths during the observation period including safety follow-up was 82 (13.7%), of which 71 patients (11.9% of FAS) died of their underlying cancer and 11 (1.8% of FAS) died of other causes not related to denosumab. Details on patient disposition by cancer type are shown in Table S1 of the online supplemental material.

### Patient demographics

**Fig. 2:**
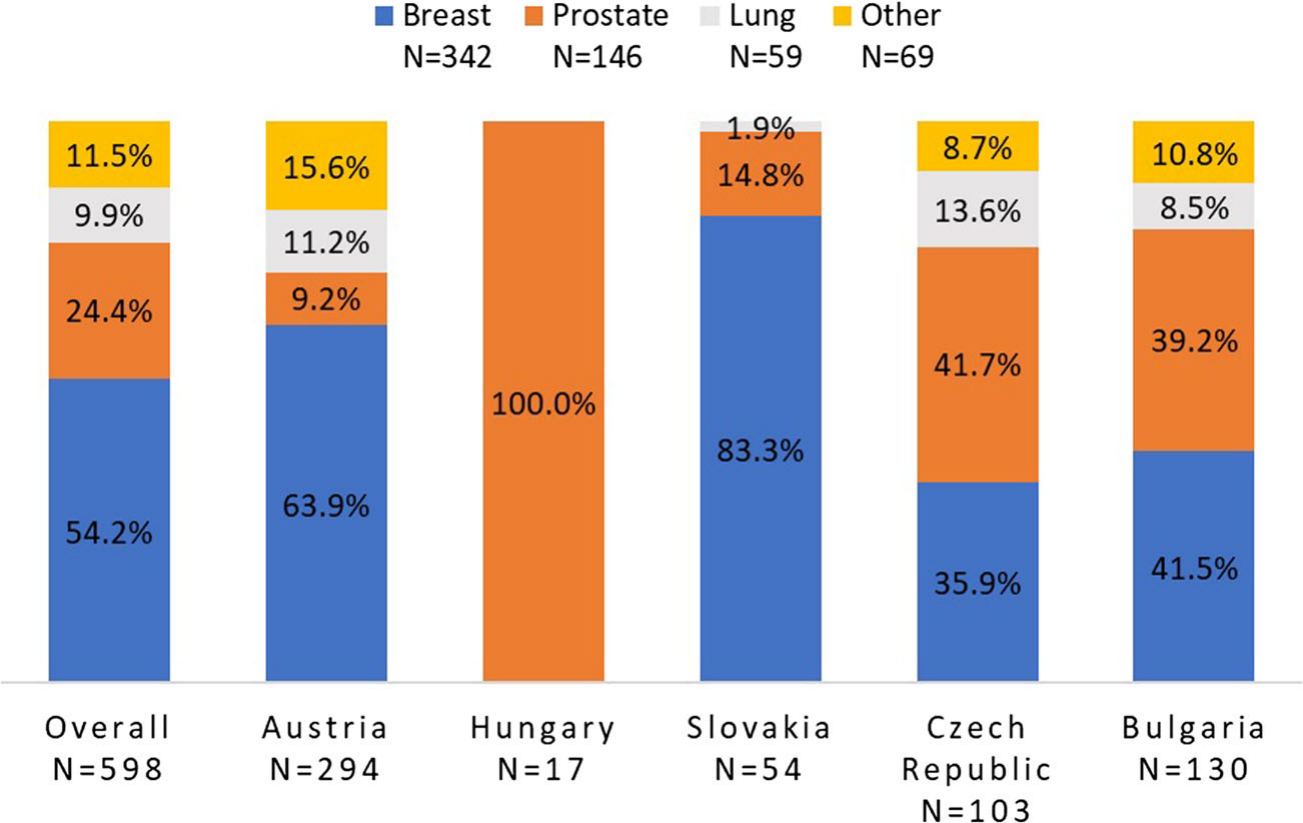
Distribution of tumor types, overall and by country (%)

Of patients, 54.2% (*n* = 324) had breast cancer, 24.4% (*n* = 146) had prostate cancer, 9.9% (*n* = 59) had lung cancer, and 11.5% (*n* = 69) had cancers summarized as “other”. Figure 2 shows the distribution of cancer types by country. Most patients were female (62.9%, *n* = 376), owing to the large number of patients with breast cancer (Table 1). The median age was 65.0 years (range 24–91); 52.2% (*n* = 312) was 65 years or older. Age differed by cancer type with the proportion of patients aged 75 or older ranging from 5.1% of patients with lung cancer (*n* = 3) to 32.2% of patients with prostate cancer (*n* = 47). ECOG performance status was 0 in 52.5% of patients (*n* = 314), 1 in 40.5% (*n* = 242), and 2 in 7.0% (*n* = 42).

**Table 1:** Patient demographics and disease characteristics

Characteristic	Breast cancer (N = 324)	Prostate cancer (*N* = 146)	Lung cancer (*N* = 59)	Other (*N* = 69)	Total (*N* = 598)
Age (years)					
Mean (SD)	60.6 (12.1)	70.5 (7.7)	62.8 (9.5)	66.3 (9.3)	63.9 (11.4)
Median (Q1, Q3)	61.0 (51.0, 70.0)	72.0 (65.0, 76.0)	65.0 (57.0, 69.0)	66.0 (60.0, 74.0)	65.0 (57.0, 72.0)
Range	30–91	46–87	24–77	46–89	24–91
Age category, *n* (%)					
< 65	197 (60.8)	32 (21.9)	27 (45.8)	30 (43.5)	286 (47.8)
≥ 65	127 (39.2)	114 (78.1)	32 (54.2)	39 (56.5)	312 (52.2)
< 75	286 (88.3)	99 (67.8)	56 (94.9)	56 (81.2)	497 (83.1)
≥ 75	38 (11.7)	47 (32.2)	3 (5.1)	13 (18.8)	101 (16.9)
Gender, *n* (%)					
Male	2 (0.6)	146 (100.0)	34 (57.6)	40 (58.0)	222 (37.1)
Female	322 (99.4)	0	25 (42.4)	29 (42.0)	376 (62.9)
ECOG status, *n* (%)					
0	194 (59.9)	70 (47.9)	22 (37.3)	28 (40.6)	314 (52.5)
1	108 (33.3)	67 (45.9)	34 (57.6)	33 (47.8)	242 (40.5)
2	22 (6.8)	9 (6.2)	3 (5.1)	8 (11.6)	42 (7.0)
Time since cancer diagnosis (months)					
*N*	322	145	59	69	595
Mean (SD)	63.9 (77.7)	30.9 (48.3)	4.7 (7.6)	39.0 (48.2)	47.1 (67.1)
Median (Q1, Q3)	35.9 (4.0, 94.5)	8.8 (2.1, 34.4)	1.8 (0.8, 4.6)	17.5 (4.8, 59.0)	19.3 (2.2, 65.0)
Range[a]	0.0–400.4	− 0.1–258.6	0.1–42.3	− 0.13–219.4	− 0.13–400.4
Missing	2	1	0	0	3
Time since cancer diagnosis category, *n* (%)					
< 1 year	105 (32.4)	79 (54.1)	54 (91.5)	25 (36.2)	263 (44.0)
1–< 2 years	25 (7.7)	15 (10.3)	3 (5.1)	14 (20.3)	57 (9.5)
2–< 5 years	77 (23.8)	28 (19.2)	2 (3.4)	14 (20.3)	121 (20.2)
5–< 10 years	60 (18.5)	13 (8.9)	0 (0)	11 (15.9)	84 (14.0)
10–< 20 years	42 (13.0)	8 (5.5)	0 (0)	5 (7.2)	55 (9.2)
≥ 20 years	13 (4.0)	2 (1.4)	0 (0)	0 (0)	15 (2.5)
Missing	2 (0.6)	1 (0.7)	0 (0)	0 (0)	3 (0.5)
Metastasis site, *n* (%)					
Bone only	128 (39.5)	113 (77.4)	20 (33.9)	18 (26.1)	279 (46.7)
Bone and other	196 (60.5)	33 (22.6)	39 (66.1)	51 (73.9)	319 (53.3)
Number of bone metastases category, *n* (%)					
1	39 (12.0)	12 (8.2)	13 (22.0)	21 (30.4)	85 (14.2)
2–4	73 (22.5)	27 (18.5)	19 (32.2)	20 (29.0)	139 (23.2)
> 4	171 (52.8)	94 (64.4)	26 (44.1)	20 (29.0)	311 (52.0)
Unknown	41 (12.7)	13 (8.9)	1 (1.7)	8 (11.6)	63 (10.5)
Non-bone metastases site[b], *n* (%)					
Liver	81 (25.0)	6 (4.1)	16 (27.1)	26 (37.7)	129 (21.6)
Lung	80 (24.7)	6 (4.1)	14 (23.7)	21 (30.4)	121 (20.2)
Brain	11 (3.4)	0 (0)	7 (11.9)	4 (5.8)	22 (3.7)
Other	96 (29.6)	28 (19.2)	23 (39.0)	24 (34.8)	171 (28.6)
Time since metastasis diagnosis (months)					
*N*	324	142	59	68	593
Mean (SD)	7.2 (22.5)	6.2 (12.0)	3.0 (6.2)	10.1 (16.8)	6.9 (18.7)
Median (Q1, Q3)	1.1 (0.5, 3.5)	1.7 (0.7, 5.8)	1.4 (0.6, 3.3)	2.7 (0.7, 12.1)	1.3 (0.6, 4.4)
Range	0.0–214.7	0.0–74.2	0.1–42.3	0.0–78.8	0.0–214.7
Missing	0	4	0	1	5
Time since metastasis diagnosis category, *n* (%)					
<1 year	287 (88.6)	124 (84.9)	57 (96.6)	50 (72.5)	518 (86.6)
1–< 2 years	15 (4.6)	6 (4.1)	1 (1.7)	9 (13.0)	31 (5.2)
2–< 5 years	15 (4.6)	10 (6.8)	1 (1.7)	6 (8.7)	32 (5.4)
5–< 10 years	3 (0.9)	2 (1.4)	0 (0)	3 (4.3)	8 (1.3)
10–< 20 years	4 (1.2)	0 (0)	0 (0)	0 (0)	4 (0.7)
≥ 20 years	0 (0)	0 (0)	0 (0)	0 (0)	0 (0)
Missing	0 (0)	4 (2.7)	0 (0)	1 (1.4)	5 (0.8)
Time since bone metastasis diagnosis (months)					
*N*	323	142	59	68	592
Mean (SD)	3.3 (12.2)	4.8 (9.0)	1.9 (3.3)	3.2 (6.8)	3.5 (10.4)
Median (Q1, Q3)	0.8 (0.3, 2.0)	1.5 (0.7, 4.6)	1.1 (0.4, 2.2)	0.8 (0.3, 2.3)	1.0 (0.4, 2.5)
Range	0.0–143.5	0.0–54.2	0.0–23.4	0.0–41.5	0.0–143.5
Missing	1	4	0	1	6
Time since bone metastasis diagnosis categories, *n* (%)					
<1 year	306 (94.4)	127 (87.0)	58 (98.3)	65 (94.2)	556 (93.0)
1–< 2 years	10 (3.1)	6 (4.1)	1 (1.7)	0 (0)	17 (2.8)
2–< 5 years	5 (1.5)	9 (6.2)	0 (0)	3 (4.3)	17 (2.8)
5–< 10 years	0 (0)	0 (0)	0 (0)	0 (0)	0 (0)
10–< 20 years	2 (0.6)	0 (0)	0 (0)	0 (0)	2 (0.3)
≥ 20 years	0 (0)	0 (0)	0 (0)	0 (0)	0 (0)
Missing	1 (0.3)	4 (2.7)	0 (0)	1 (1.4)	6 (1.0)
Diagnosis method of bone metastasis, *n* (%)					
By symptoms	81 (25.0)	35 (24.0)	21 (35.6)	17 (24.6)	154 (25.8)
Asymptomatic/imaging	237 (73.1)	111 (76.0)	38 (64.4)	50 (72.5)	436 (72.9)
Unknown	6 (1.9)	0 (0)	0 (0)	2 (2.9)	8 (1.3)

Percentages are based on the number of patients in full analysis set

[a] Negative values are from the following two patients: Patient 1, first XGEVA dose: 2014-07-16, cancer diagnosis date: 2014-07-18. Patient 2, first XGEVA dose: 2015-11-05, cancer diagnosis date: 2015-11-09

[b] Percentages in this section may add up to more than 100% because one patient may have different metastasis sites

### Disease characteristics

The median (Q1, Q3) time since cancer diagnosis was 19 months (2.2, 65.0) with 44.0% of patients (*n* = 263) receiving the diagnosis for cancer less than 1 year before enrollment. The median time since diagnosis ranged from 1.8 months in lung cancer patients to 35.9 months in breast cancer patients (Table 1). Patients were required to have confirmed metastatic disease. The median time since diagnosis of metastatic disease was 1 month (IQR 0.6, 4.4), and in 86.6% of patients (*n* = 518) metastatic disease was diagnosed less than 1 year before enrollment. By metastasis site, 46.7% (*n* = 279) had bone metastases only and 53.3% (*n* = 319) had metastases in the bone and other sites. Other metastatic sites were in the liver in 21.6% of patients (*n* = 129), lung in 20.2% (*n* = 121), brain in 3.7% (*n* = 22), and other sites in 28.6% (*n* = 171). Patient could have metastases in more than one site. Bone metastases were diagnosed less than 1 year before enrollment in 93.0% of patients (*n* = 556) and were mostly asymptomatic and diagnosed by imaging (72.9%, *n* = 436; Table 1). The median time between diagnosis of bone metastases and initiation of denosumab was 1.3 months (IQR 0.6, 4.4).

### Prior skeletal-related events

Prior to enrollment, SREs were confirmed in 10.9% of patients (*n* = 65): 7.5% (*n* = 45) had pathological fractures, 2.2% (*n* = 13) required radiation to the bone, 1.5% (*n* = 9) had surgery to the bone, and 0.5% (*n* = 3) had spinal cord compression. The time between diagnosis of an SRE and enrollment was less than 3 months in 7.4% of patients (*n* = 44) and between 3 and 6 months in 2.3% (*n* = 14); in 2 patients (0.3%), it was between 6 and 12 months, and in 5 patients (0.8%), it was longer than 12 months.

### Anticancer therapies

In the metastatic setting, 35.3% of patients (*n* = 211) had received chemotherapy prior to starting denosumab, 32.8% (*n* = 196) had received previous endocrine therapy, 14.4% (*n* = 86) radiotherapy, and 7.9% (*n* = 47) surgery. During the study observation period and concomitantly with denosumab, 52.3% of patients (*n* = 313) received chemotherapy, 46.3% (*n* = 277) endocrine therapy, 15.7% (*n* = 94) radiotherapy, and 3.5% (*n* = 21) surgery.

### Denosumab treatment rationale and duration

Prior to starting denosumab, 7.7% of patients (*n* = 46) had received other antiresorptive agents. Previous antiresorptive therapies were zoledronic acid (6.2%, *n* = 37), ibandronate (0.3%, *n* = 2), pamidronate (0.3%, *n* = 2), and unspecified others (0.8%, *n* = 5). Antiresorptive agents were mainly administered intravenously (6.7%, *n* = 40); 1.0% (*n* = 6) received them per os. All 46 patients received their antiresorptive therapy for 6 months or less, as per inclusion criteria. Reasons for not continuing previous antiresorptive therapies were intolerability in 1.5% (*n* = 9), patient’s wish (0.5%, *n* = 3), or physician decision (5.7%, *n* = 34). Physicians decided to stop the previous antiresorptive agents because of the route of administration (3.3%, *n* = 20), renal insufficiency (1.5%, *n* = 9), or unspecified other reasons (1.2%, *n* = 7).

The most frequent physician-reported reasons for the choice of denosumab were the prevention of first SRE (63.5%, *n* = 380; first most important), superior efficacy of denosumab (28.3%, *n* = 169, second most important), and better safety profile of denosumab (15.6%, *n* = 93; third most important).

The patients received a median (Q1, Q3) of 11 doses (6.0, 12.0) of denosumab over a period of 309 days (168.0, 319.0).

### Calcium and vitamin D supplementation

The median (Q1, Q3) serum calcium level at enrollment was 2.35 (2.25, 2.44) mmol/L. At the second dose of denosumab, the serum calcium level reached a nadir at 2.26 (2.15, 2.37) mmol/L. Serum calcium remained above this lowest value from the third dose onwards throughout the study. At enrollment, 70.2% of patients (*n* = 420) received calcium supplementation and 71.4% (*n* = 427) received vitamin D supplementation. This proportion increased to approximately 80% at dose 2 and steadily decreased thereafter (Fig. S1, online supplemental material).

### Persistence at 24 weeks (primary outcome measure)

**Fig. 3:**
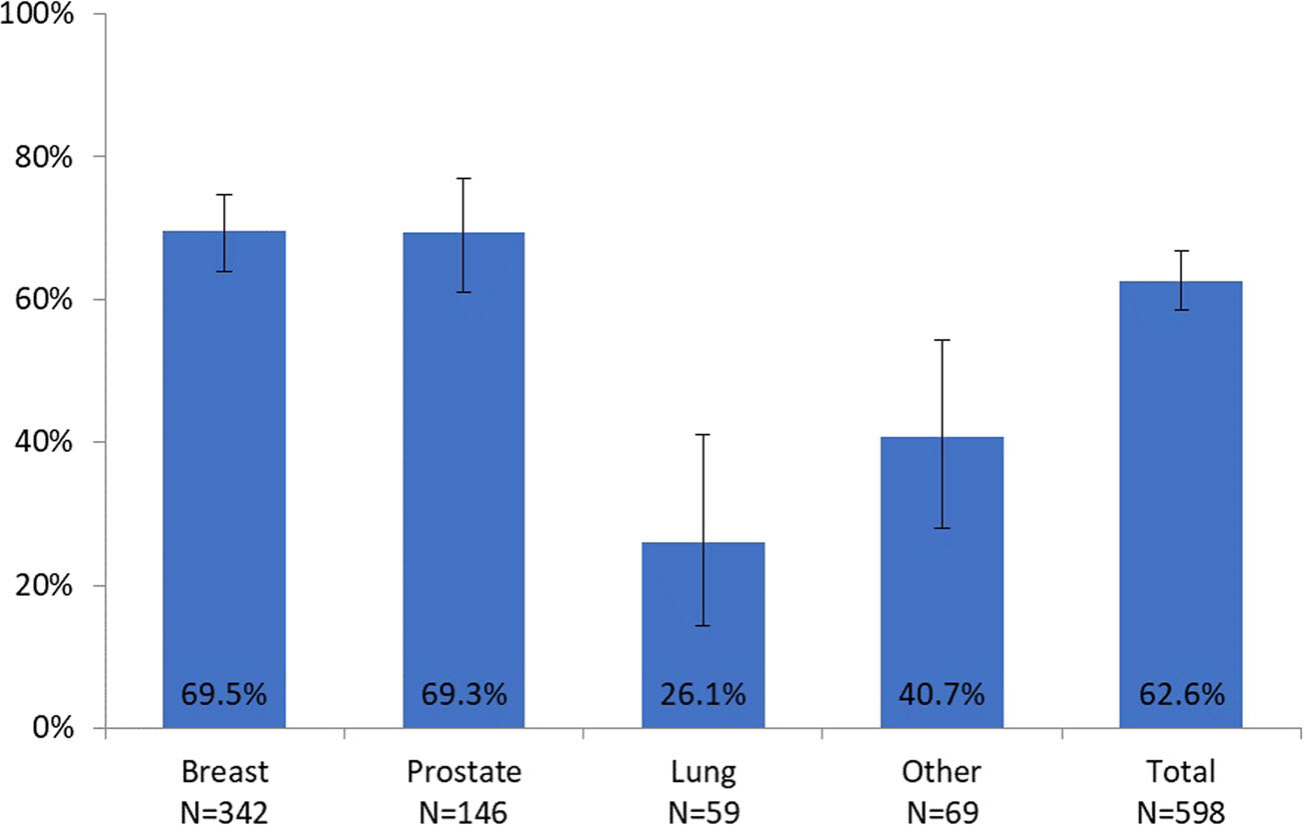
Persistence for denosumab at 24 weeks, overall and by tumor type (%, 95% CI)

**Fig. 4:**
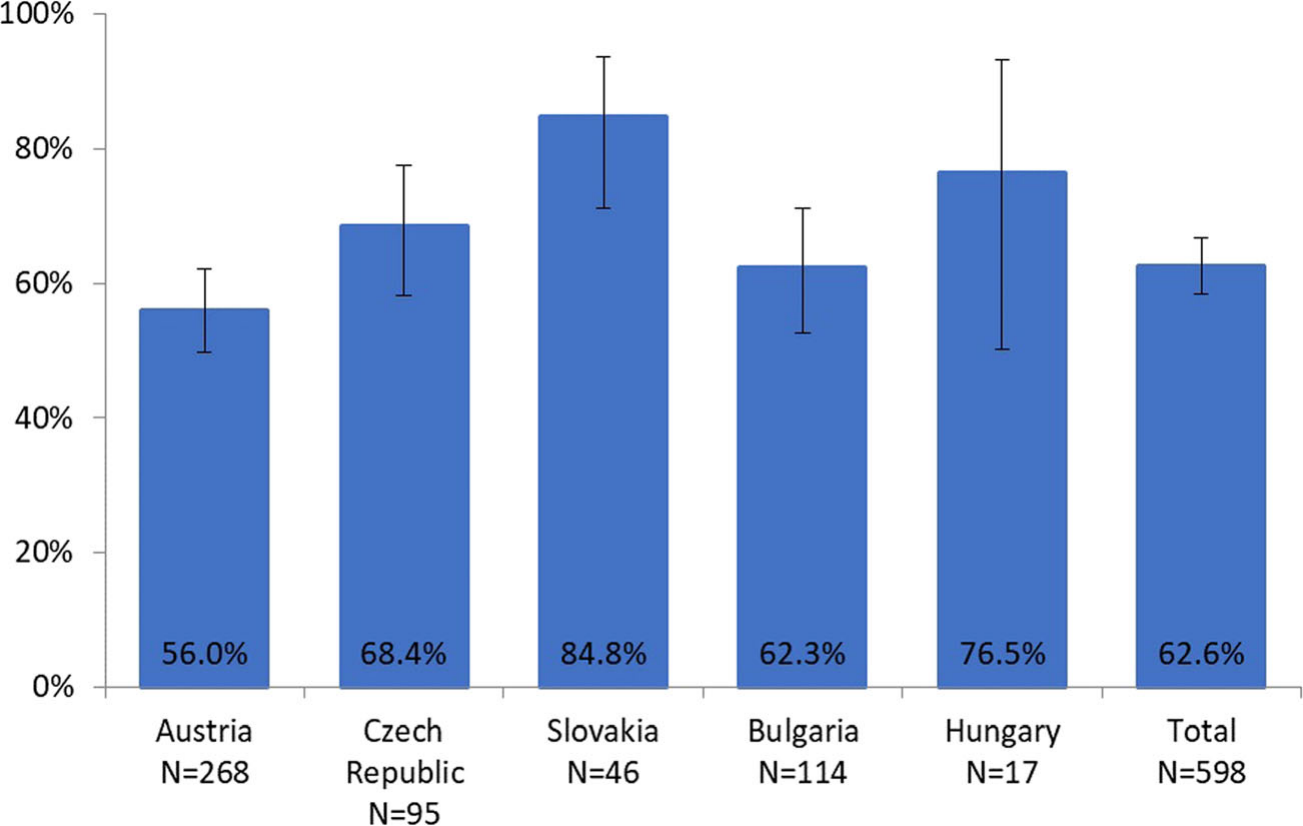
Persistence for denosumab at 24 weeks, overall and by country (%, 95% CI)

Persistence at week 24 was 62.6% (95% CI 58.4, 66.7) overall, and ranged between 26.1% for lung cancer and 69.5% for breast cancer, and between 56.0% for Austria and 84.8% for Slovakia. Figures 3 and 4 show persistence at 24 weeks by tumor type and by country, respectively.

Persistence differed by tumor type, impacting on persistence in the countries. In Austria and Slovakia, the largest group of represented tumors was breast cancer; in Hungary, only prostate cancer patients were enrolled. In the Czech Republic and Bulgaria, breast and prostate cancer patients were almost equally represented and formed the largest groups (Fig. 2).

### Persistence at 48 weeks

Persistence at 48 weeks was 40.1% (95% CI 35.9, 44.4). Patterns by tumor type and by country were similar to the persistence results for week 24.

### Time to non-persistence

The Kaplan-Meier (KM) median (95% CI) time to non-persistence was 274.0 (232.0, 316.0) days, with 317.0 (263.0, 335.0) in breast cancer, 325.0 (271.0, 344.0) in prostate cancer, 118.0 (59.0, 144.0) in lung cancer, and 118.0 (57.0, 230.0) in other cancers.

An analysis of median time to non-persistence by previous antiresorptive therapy (y/n) showed a KM median (95% CI) of 294.0 days (168.0, 344.0) for 46 patients with previous antiresorptive therapy and 273.0 days (232.0, 316.0) for 552 patients with no previous antiresorptive therapy.

Using a Cox proportional hazards model, tumor type (breast versus other, prostate versus other), previous antineoplastic therapy (y/n), and ECOG status (0 versus 2) were found to be significantly associated with time to non-persistence with denosumab (all factors with *p* < 0.05, Wald test).

### Reasons for non-persistence

**Fig. 5:**
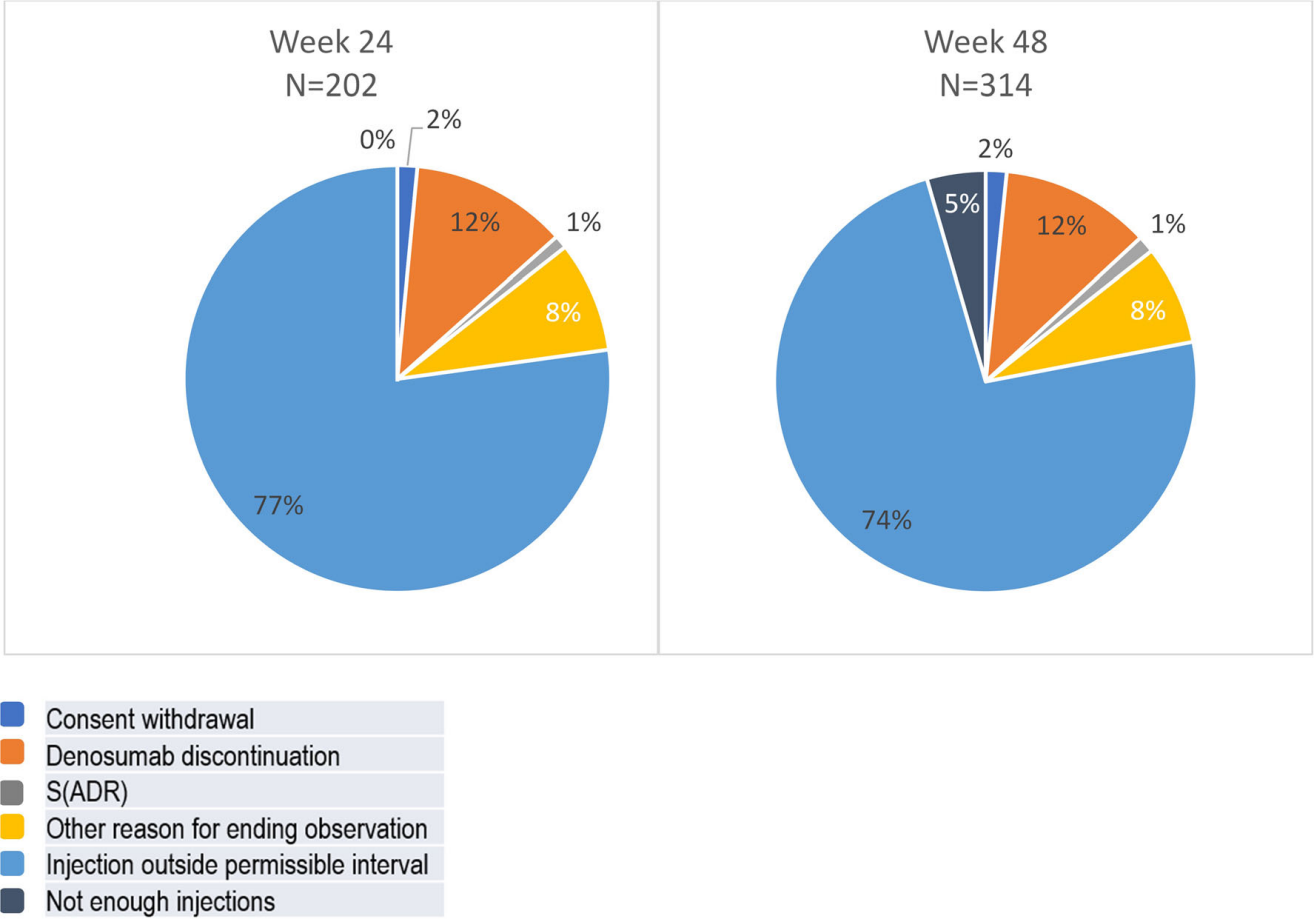
Reasons for non-persistence for denosumab at 24 weeks, overall and by country (%) (S)ADR, (serious) adverse drug reaction; permissible time windows between injections of a maximum of 35 days were defined by the protocol

Documented reasons for non-persistence were premature termination of denosumab therapy, an ADR, withdrawal of informed consent, an insufficient number of injections (week 48 only), and unspecified other reasons. Figure 5 shows reasons for non-persistence at weeks 24 and 48. Missing permissible injection intervals were documented as the most frequent reason for non-persistence at week 24 as well as week 48 (see “Methods” section for definitions). In the sensitivity analysis (see online supplement for methods), extending the permissible time windows between injections and week 24 and 48, respectively, as described earlier, increased the proportion of persistent patients by more than 10%.

### Pain management

**Fig. 6:**
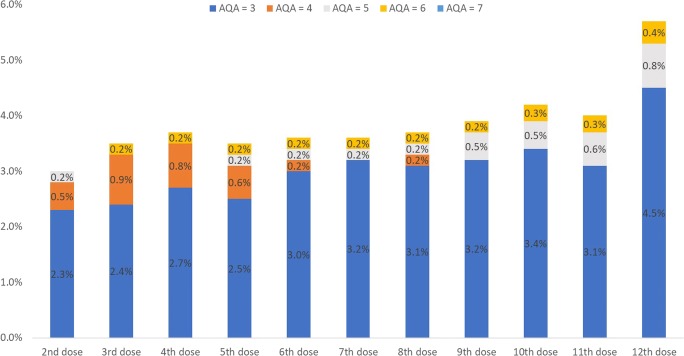
Proportion of patients with AQA score ≤ 2 at baseline shifting to AQA score > 2 at later denosumab doses

Overall, the requirements for strong analgesics were generally low. The proportion of patients not requiring any analgesics remained stable at approximately 60% of patients with available values at the respective timepoints. When receiving pain medication at baseline, most patients received non-opioid analgesics (20.2%, *n* = 121; AQA score 1) or strong opioids at a low daily dose of < 75 mg oral morphine equivalents (11.4%, *n* = 68; AQA score 3). When assessing the shift in analgesic use among patients with no or weak opioid analgesics (AQA score ≤ 2) at baseline, very few patients shifted to an AQA category > 2 (i.e., strong opioids at increasing doses) at later timepoints (Fig. 6).

### Safety

Only ADRs considered related to denosumab by the treating physician were collected. Overall, 10.2% of patients (*n* = 61) experienced an ADR. The most frequently reported ADR was hypocalcemia (7.4%, *n* = 44). Ten patients (1.7%) experienced ADRs leading to discontinuation of denosumab. Serious ADRs were documented for 1.3% of patients (*n* = 8). ONJ was documented in 0.7% of patients (*n* = 3). The median (IQR) duration to first ONJ event based on these three patients was 165.0 days (105.0, 298.0). Other reported serious ADRs were costovertebral angle tenderness, pain, hypocalcemia, peripheral edema, dyspnea, swelling face, and cellulitis (*n* = 1, respectively). No fatal ADRs occurred. Overall, the exposure-adjusted incidence rate was 0.187 (95% CI 0.147, 0.235) for ADRs per 100 patient-years and 0.027 (95% CI 0.014, 0.049) for serious ADRs.

## Discussion

In the present study, 62.6% of patients demonstrated regular and continuous use of denosumab, i.e., persistence, over 24 weeks and 40.1% over 48 weeks. The most frequent reason for non-persistence was a delay in drug administration. Only 4.8% of patients stopped denosumab treatment following the decision of their treating physician. In the similarly designed German X-TREME study, the final analysis on 1008 patients, included in the persistence assessment, showed persistence with denosumab at week 24 of 61.5%. Persistence at week 48 was 37.7%. These findings are very similar to the results of the present study. The proportion of patients persistent with denosumab at 24 weeks was previously assumed at 60% based on phase III studies [6, 9–11]. The observed persistence at 24 weeks found in this study (62.6%) was thus very similar to previous estimates.

In a retrospective analysis of a German sick fund claims database including 1156 adult patients with solid tumors newly diagnosed with bone metastases and receiving denosumab or bisphosphonates, persistence was defined as continuous prescriptions with < 90-days gaps. Of patients with breast, prostate, and lung cancer, respectively, 25%, 17%, and 20% had prior SREs. For breast cancer, persistence at 1 year, according to the above definition, was 78% for denosumab and 58%, 56%, and 54% for ibandronate, pamidronate, and zoledronate, respectively. For prostate cancer, persistence with denosumab and zoledronate were 58% and 50%, respectively. Finally, in lung cancer, persistence for denosumab, pamidronate, and zoledronate was 68%, 34%, and 60%, respectively. Persistence was lower in a sensitivity analysis in which the definition of persistence was stricter, applying 60-day gaps/windows. The definition of persistence differed between these studies and was substantially stricter in the present study because the German sick fund study did not consider persistence for each of the individual once in a month administrations and missing the recommended administration interval once was the most important reason for non-persistence.

The incidence of osteonecrosis was 0.5% in the present study (*n* = 3), with two confirmed cases of ONJ and one with unspecified location. In the German X-TREME study, 15 patients with suspected ONJ (1.3%) were reported. In randomized controlled studies of denosumab, 2% of breast cancer patients, 2.3% of prostate cancer patients, and 1.1% of patients with either a solid tumor or multiple myeloma experienced ONJ [9–11].

This was an observational study with all limitations inherent to the study design, especially selection and reporting bias, and lack of blinding and of a control group. Persistence was estimated taking all drop-outs related to denosumab into account. Impact of bias was addressed in sensitivity analyses. The extension of the allowed time windows was the only factor that had an impact on results in the sensitivity analyses. Importantly, missing the recommended administration interval was also the most important reason for non-persistence. The sensitivity analyses revealed that the different ways of handling drop-outs did not change the results of the primary and secondary outcome measure. The subgroup analyses by tumor type and by country revealed some differences in persistence, which limit generalizability of the results to other tumors, countries, and regions. An analysis of the distribution of tumor types by country revealed that differences between countries can at least in part be explained by the different distributions of tumors in each country. Another difference between countries is the method of dispensation of denosumab to the patients. In Austria, denosumab was mainly dispensed as a retail product at the time of study; in Hungary, it was available as a retail product and reimbursed in prostate cancer only. In Czech Republic and Bulgaria, it was administered exclusively in hospitals. In Slovakia, it was a retail product during the first part of the study and a hospital product as of October 2016. Patients received a diary to report each administration of denosumab. Especially in countries where denosumab was distributed as a retail product and not exclusively administered in the hospital, patient self-reporting may be prone to inaccuracies.

## Conclusions

The majority of patients were persistent with a once in a month administration of denosumab for over 24 weeks. The type of primary tumor, previous antineoplastic therapy, and ECOG status appeared to influence persistence. The most frequent reason for non-persistence was the violation of administration intervals. Most patients reported taking calcium and vitamin D supplementation as recommended as per label. The incidence of ADRs, especially of ONJ, was in line with expectations from previous studies.

## Electronic supplementary material

ESM 1 (DOCX 29 kb)

## Data Availability

Amgen holds the source data and authors had access to the data. Qualified researchers may request data from Amgen clinical studies. Complete details are available at the following: http://www.amgen.com/datasharing.
