# Supplementary material for: Prospective observational study to evaluate the persistence of treatment with denosumab in patients with bone metastases from solid tumors in routine clinical practice: final analysis
Source: Support Care Cancer. 2019 Jul 26;28(4):1855–65. doi: 10.1007/s00520-019-04988-7 (PMC7036060; doi:10.1007/s00520-019-04988-7)
Supplement: Supplementary file 1 — (DOCX 29 kb) [file 520_2019_4988_MOESM1_ESM.docx]

**Online supplemental material**

**Prospective observational study to evaluate the persistence of treatment with denosumab in patients with bone metastases from solid tumors in routine clinical practice: final analysis**

Ferdinand Haslbauer, Andreas Petzer, Martin Safanda, Antoaneta Tomova, Miriam Porubska, Zoltán Bajory, Daniela Niepel, Christine Jaeger, Katja Bjorklof, Dmitry Kalinin, Richard Greil

**Methods used for sensitivity analyses**

Sensitivity analyses were performed to assess the impact of patients who died or were lost to follow-up before the appropriate study endpoint. For the analysis of persistence at 24 weeks, patients who died or were lost to follow-up prior to week 24 were excluded in any case irrespective of whether they violated the persistence definition above before the discontinuation date. The same was repeated for the endpoint at week 48.

Additionally, sensitivity analyses with extended time windows were performed. The analyses repeated the definition for persistence with the following exceptions:

1. Extended time window after 24 and 48 weeks endpoints:

- a time window of +2 weeks was allowed after the 24 weeks endpoint.
- a time window of +7 weeks was allowed after the 48 weeks endpoint.

2. Extended time window between the injections and after 24 and 48 weeks endpoints:

- an additional time window of 14 days (instead of 7 days) was allowed for each injection relative to the previous injection for persistence to denosumab at 24 weeks and after 48 weeks.
- a time window of +4 weeks was allowed after the 24 weeks endpoint.
- a time window of +10 weeks was allowed after the 48 weeks endpoint.

**Table S1. Patient disposition**

|  | Breast cancer (N=324) n (%) | Prostate cancer (N=146) n (%) | Lung cancer (N=59) n (%) | Other (N=69) n (%) | Total (N=598) n (%) |
| --- | --- | --- | --- | --- | --- |
| Number of patients in FAS[a] | 324 (100.0%) | 146 (100.0%) | 59 (100.0%) | 69 (100.0%) | 598 (100.0%) |
|  | | | | | |
| Number of patients who completed 24 weeks of observation | 260 (80.2%) | 123 (84.2%) | 29 (49.2%) | 39 (56.5%) | 451 (75.4%) |
| Number of patients who discontinued before week 24 | 64 (19.8%) | 23 (15.8%) | 30 (50.8%) | 30 (43.5%) | 147 (24.6%) |
| Reason for discontinuation: |  |  |  |  |  |
| Death | 25 (7.7%) | 10 (6.8%) | 14 (23.7%) | 10 (14.5%) | 59 (9.9%) |
| Lost to follow-up | 10 (3.1%) | 4 (2.7%) | 5 (8.5%) | 7 (10.1%) | 26 (4.3%) |
| ICF withdrawal | 3 (0.9%) | 1 (0.7%) | 0 | 1 (1.4%) | 5 (0.8%) |
| Denosumab discontinuation | 16 (4.9%) | 5 (3.4%) | 6 (10.2%) | 8 (11.6%) | 35 (5.9%) |
| (S)ADR | 0 | 1 (0.7%) | 0 | 1 (1.4%) | 2 (0.3%) |
| Other | 10 (3.1%) | 2 (1.4%) | 5 (8.5%) | 3 (4.3%) | 20 (3.3%) |
|  | | | | | |
| Number of patients who completed 48 weeks of observation | 227 (70.1%) | 111 (76.0%) | 20 (33.9%) | 29 (42.0%) | 387 (64.7%) |
| Number of patients who discontinued before week 48 | 97 (29.9%) | 35 (24.0%) | 39 (66.1%) | 40 (58.0%) | 211 (35.3%) |
| Reason for discontinuation: |  |  |  |  |  |
| Death | 35 (10.8%) | 14 (9.6%) | 18 (30.5%) | 13 (18.8%) | 80 (13.4%) |
| Lost to follow-up | 14 (4.3%) | 6 (4.1%) | 6 (10.2%) | 9 (13.0%) | 35 (5.9%) |
| ICF withdrawal | 4 (1.2%) | 1 (0.7%) | 1 (1.7%) | 1 (1.4%) | 7 (1.2%) |
| Denosumab discontinuation | 28(8.6%) | 9 (6.2%) | 8 (13.6%) | 11 (15.9%) | 56 (9.4%) |
| (S)ADR | 2 (0.6%) | 2 (1.4%) | 0 | 1 (1.4%) | 5 (0.8%) |
| Other | 14 (4.3%) | 3 (2.1%) | 6 (10.2%) | 5 (7.2%) | 28 (4.7%) |
|  | | | | | |
| Number of patients who completed safety follow-up[b] | 269 (83.0%) | 125 (85.6%) | 33 (55.9%) | 47 (68.1%) | 474 (79.3%) |
| Number of patients who did not complete safety follow-up[b] | 6 (1.9%) | 1 (0.7%) | 2 (3.4%) | 0 | 9 (1.5%) |
| Reason for discontinuation: |  |  |  |  |  |
| Death | 1 (0.3%) | 0 | 1 (1.7%) | 0 | 2 (0.3%) |
| Lost to follow-up | 2 (0.6%) | 1 (0.7%) | 0 | 0 | 3 (0.5%) |
| ICF withdrawal | 0 | 0 | 0 | 0 | 0 |
| Other | 3 (0.9%) | 0 | 1 (1.7%) | 0 | 4 (0.7%) |
|  | | | | | |
| Number of patients who died | 36 (11.1%) | 14 (9.6%) | 19 (32.2%) | 13 (18.8%) | 82 (13.7%) |
| Death reason: |  |  |  |  |  |
| Cancer-related | 34 (10.5%) | 10 (6.8%) | 16 (27.1%) | 11 (15.9%) | 71 (11.9%) |
| Other | 2 (0.6%) | 4 (2.7%) | 3 (5.1%) | 2 (2.9%) | 11 (1.8%) |
|  | | | | | |
| Death related to denosumab?[c] |  |  |  |  |  |
| No | 2 (0.6%) | 4 (2.7%) | 3 (5.1%) | 2 (2.9%) | 11 (1.8%) |
| Yes | 0 | 0 | 0 | 0 | 0 |
|  | | | | | |
| Continuing denosumab treatment after EOT? |  |  |  |  |  |
| No | 19 (5.9%) | 3 (2.1%) | 7 (11.9%) | 6 (8.7%) | 35 (5.9%) |
| Yes | 221 (68.2%) | 112 (76.7%) | 18 (30.5%) | 28 (40.6%) | 379 (63.4%) |
| Unknown | 7 (2.2%) | 2 (1.4%) | 2 (3.4%) | 2 (2.9%) | 13 (2.2%) |
|  | | | | | |
| Reason for denosumab treatment discontinuation: |  |  |  |  |  |
| (S)ADR | 3 (0.9%) | 2 (1.4%) | 1 (1.7%) | 2 (2.9%) | 8 (1.3%) |
| Physician’s decision | 13 (4.0%) | 3 (2.1%) | 10 (16.9%) | 3 (4.3%) | 29 (4.8%) |
| Patient’s decision | 16 (4.9%) | 4 (2.7%) | 2 (3.4%) | 6 (8.7%) | 28 (4.7%) |
| Switch to other antiresorptive treatment | 3 (0.9%) | 0 | 1 (1.7%) | 1 (1.4%) | 5 (0.8%) |
| Other | 12 (3.7%) | 3 (2.1%) | 1 (1.7%) | 5 (7.2%) | 21 (3.5%) |

[a] 634 patients were enrolled; 319 from Austria, 130 from Bulgaria, 109 from the Czech Republic, 58 from Slovakia, and 18 from Hungary. 36 patients were excluded from the analysis. The reasons for exclusion from analysis were violation of inclusion or exclusion criteria, erroneous double entry in the database, or entry by mistake, e.g. erroneously entering a training data set in the real database instead of the training database.

[b] Only patients who did not die and weren’t lost to follow-up are included in this section.

[c] Only patients with death reason ‘other’ are included in this section.

Figure S1. Serum calcium levels, and calcium and vitamin D supplementation over time
